# Identification and Description of the Key Molecular Components of the Egg Strings of the Salmon Louse (*Lepeophtheirus salmonis*)

**DOI:** 10.3390/genes10121004

**Published:** 2019-12-03

**Authors:** Andreas Borchel, Heidi Kongshaug, Frank Nilsen

**Affiliations:** SLRC-Sea Lice Research Centre, Department of Biological Sciences, University of Bergen, Bergen 5020, Norway

**Keywords:** reproduction, egg attachment, RNAi, Crustacean, Copepod, structure, cement, EGF, TSP, invertebrate

## Abstract

The salmon louse *Lepeophtheirus salmonis* is a parasite of Atlantic salmon and other salmonids. Every year, it causes high costs for the Norwegian aquaculture industry. While the morphology of the female genital tract has been described, knowledge of the molecular basis of reproduction is very limited. We identified nine genes which are expressed exclusively in the female cement gland, the organ responsible for cement production, which is used to hold the eggs together and keep them attached to their mother in egg strings. Six of these genes encode proteins with signal peptides and probably form the main component of the cement. Two other genes are peroxidases, which are probably important in the cement formation. The last gene is not similar to any known protein, but contains a transmembrane domain. A knockdown of all these genes leads to missing or deformed egg strings, preventing reproduction of the lice. The correct assemblage of the cement in the cement gland is essential for successful reproduction of salmon lice. Similar proteins seem to be present in other copepod species, as well.

## 1. Introduction

The salmon louse *Lepeophtheirus salmonis* (Krøyer 1837) belongs to the order of Siphonostomatoida within the subclass of Copepoda, which are part of the Crustaceans [1]. This species is a parasite of Salmonids in general, and especially of the Atlantic salmon, and feeds on the blood, mucus, and skin of its host [2]. The frequent occurrence of salmon lice in salmon cages renders them a big problem in salmonid aquaculture and causes high annual costs in this industrial sector [3,4]. Salmon lice undergo a life cycle consisting of eight life stages that are separated by moult—the shedding of the old cuticle [5].

**Figure 1:**
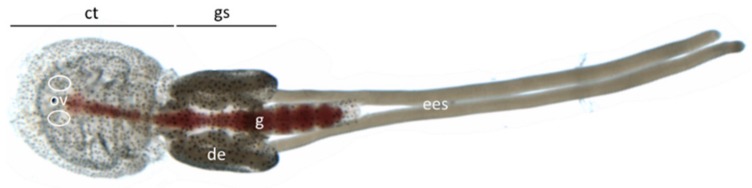
Anatomy of an adult female salmon louse. The body can be divided into cephalothorax (ct) and genital segment (gs). Ovaries (ov) are not visible, but their location is marked with white circles. The blood-filled gut (g) is reaching from the mouth opening throughout the gs. Developing eggs (de) are filling up the genital segment. The extruded egg strings (ees) are longer than the animal itself.

Adult females produce eggs in the paired ovaries, which are located in the cephalothorax, close to the eyes (Figure 1). The oocytes move through the oviducts into the genital segment [6], where the oviducts widen and the eggs become loaded with vitellogenins [7] and lipids [8]. Production of the vitellogenins LsVit1, LsVit2 [7] and the yolk-associated protein LsYAP [9], necessary for successful egg maturation, take place in the subcuticular tissue of the lice. Egg development and maturation is controlled by the TOR pathway, including the upstream regulator LsRheb [10], as well as by the ecdysone receptor LsECR [11]. 

When ready, the eggs are extruded from the oviducts into sac-like structures, called egg strings. During the extrusion, the eggs become fertilized by sperm cells that are stored in a spermatheca in the female upon mating with a male. Additionally, the eggs become embedded in cement, which is produced by the “sausage-shaped” vitellaria or cement glands [12] in the genital antra. The female carries the egg strings attached to its body with a muscularly controlled hook apparatus until the offspring hatches [12]. However, this attachment is only mechanical; the egg strings develop also after removal from the females, a property utilized for the culture and breeding of salmon lice [13].

The number of eggs in, and the length of, the egg string is lower in the first string compared to subsequent strings [2] and is dependent on water temperature [14]. Frequency of egg-string extrusion is also dependent on temperature [15]. 

The structure and morphology of the female reproductive system [6] and the hooking apparatus [12] have been described in detail; however, knowledge of key components of egg string formation is still limited. The egg string might be a target for pesticide development. Crustacean early life stages might have the highest stress sensitivity [16], and using them as a point of attack might help to control a complete salmon louse population on a fish farm.

In this study, we identify genes that are expressed in the genital segment of adult females. We show that these genes are expressed in the cement glands and that the encoded proteins are the building blocks of the egg string.

## 2. Materials and Methods

### 2.1. Candidate Gene Identification

We screened the Licebase (https://licebase.org) RNA-Seq database for genes with distinct gene-expression patterns. Licebase contains gene expression data for, among others, unfertilized egg strings (located within the genital segment), egg strings 0–24 h after fertilization (extruded), and egg strings 2–7 days after fertilization. It was very striking that some of the genes, which were very strongly expressed in unfertilized egg-strings, were not expressed in fertilized egg strings at all. We searched for genes that showed this specific expression pattern with a strong expression in unfertilized egg strings and low or absent expression in egg strings 0–24 h after fertilization. To identify these highly expressed genes in unfertilized egg strings, we employed the following search criteria: CPM value over 100 in unfertilized egg string and less than 1% of the unfertilized egg string’s CPM value in fertilized egg strings after 0–24 h, 2–7 days, and Nauplius I larvae.

To get a first idea of the genes’ function, the predicted protein sequences were blasted (blastp) against the NCBI nonredundant protein database [17].

### 2.2. Animal Culture

Salmon lice were maintained in the laboratory. Egg strings, nauplii, and copepodids were kept in individual wells in a flow-through system [5]. Later stages were kept on farmed Atlantic salmon (*Salmo salar*) at 10 °C. All experiments were in accordance with Norwegian animal welfare legislation. Animals needed for experiments were sampled and put directly into RNAlater (Thermo Fisher Scientific, Waltham, USA). They were kept at 4 °C overnight and then stored at −20 °C until RNA purification.

### 2.3. RNA Extraction and cDNA Synthesis

RNA was isolated from whole animals stored in RNAlater using TRI Reagent (Sigma-Aldrich, St. Louis, USA), following the manufacturer’s protocol. Animals were transferred from RNAlater to 1 mL of TRI reagent. To lyse animals in a TissueLyser II bead mill (Qiagen, Hilden, Germany), zirconium oxide beads with a diameter of 1.4 mm were used for nauplius, copepodids, and chalimus, and 5 mm stainless steel beads were used for preadult and adult lice. Homogenization took place at maximal speed for 3 min. Afterward, 200 μL of chloroform was added. After a centrifugation step of 15 min at 4 °C at maximum speed, the colorless upper phase was mixed with the same volume of ispopropanol. The RNA pellet was washed two times with 75% ethanol, dried, and then reconstituted in 50–100 μL nuclease-free water. RNA concentration and quality (260/280; 260/230) were determined on a Nanodrop ND-1000 spectrophotometer (Thermo Scientific, Waltham, USA). All RNA was DNase-treated (Thermo Fisher Scientific, Waltham, USA), employing 1 μg of RNA in a 10 μL reaction, for 15 min, at room temperature. The reaction was stopped by adding 1 μL of 25 mM EDTA and performing a 10 min incubation at 65 °C.

Complementary DNA (cDNA) was synthesized in 10 μL reactions, using the AffinityScript qPCR cDNA synthesis Kit (Agilent, Santa Clara, USA), employing a 2:1 ratio of OligodT and Random hexamer primers and 2 μL of DNase-treated RNA. The samples were incubated at 25 °C for 5 min, followed by 30 min at 42 °C and 5 min at 95 °C. The resulting cDNA was then diluted to 1:10 and stored at −20 °C, until further use.

### 2.4. Gene Sequencing and Characterization

To validate the gene sequences obtained from Licebase, we performed PCR reactions, spanning the known sequences, as well as 5’ and 3’ RACEs. Initial PCRs were performed by using the GoTaq G2 Flexi DNA Polymerase (Promega, Madison, USA). The rapid amplification of cDNA ends (RACE) was performed by using a SMARTer RACE cDNA Amplification kit (Clontech, Mountain View, USA), following the manufacturers’ instructions. After identification of start and stop codon of the longest open reading frame, employing NCBI’s ORFfinder tool (https://www.ncbi.nlm.nih.gov/orffinder/), and checking a meaningful translation, using SmartBlast (https://blast.ncbi.nlm.nih.gov/smartblast/), primers (Table S1) spanning the complete CDS were designed, and the complete CDS was amplified by using the high fidelity Q5-polymerase (NEB, Ipswich, USA). PCR products were cleaned up by using ExoSAP-IT Express (Affymetrix, Santa Clara, USA) and sequenced by using BigDye Terminator v3.1, on a 3730XL Analyzer (Applied Biosystems, Foster City, USA), located at the sequencing facility at the University of Bergen. The sequences were blasted (blastn) [17] against the *L. salmonis* genome assembly, to determine to which genes the transcript belonged; they were then analyzed for the open reading frame, and the translation was checked for conserved domains, using InterProScan [18]. 

### 2.5. Quantitative PCR

Gene expression was determined using by PowerUp SYBR Green Master Mix (Thermo Fisher Scientific, Waltham, USA), containing specific concentrations of magnesium and dNTPs and polymerase chosen by the manufacturer, on QuantStudio 3 qPCR machines (Applied Biosystems, Foster City, USA). The reaction size was 10 µl, including 2 µl of cDNA. The final primer concentration was 0.5 µM each. Primers are given in Table S1 and were placed either on exon–intron boundaries or on two exons spanning an intron, to prevent amplification of potentially present genomic DNA. The specificity of the primers was checked using a blast-n search in Licebase on the salmon louse genome.

The thermocycling parameters were as follows: initiation, 50 °C for 2 min; holding, 95 °C for 2 min; 40 cycles of 95 °C for 15 seconds; and then 60 °C for 1 min. Melting curves were generated after every qPCR run and checked for the presence of only one specific peak for each gene in each sample. Every plate run contained non-template controls for each master mix run on each plate, making sure that there was no contamination of the reagents. All samples were run in technical duplicates, on the same plate. Ct values were generated by the QuantStudio Design & Analysis Software 1.5.1 (Applied Biosystems, Foster City, USA), employing a threshold of 0.2 on all plates, and the average of the Ct values from the technical duplicates were used for further calculation. Using relative standard curves and the deduced reaction efficiencies (Table S1), the relative gene expression in relation to the reference genes Elongation factor 1α (*EF1A*) and Adenine Nucleotide Translocator 3 (*ADT3*) was calculated. Both reference genes were established, validated as stable, and used for the analysis of gene expression throughout the development of salmon lice before [19,20,21]. Calculations were performed in Microsoft Excel 2016.

To determine gene expression during different life stages, animals from all eight life stages were collected. Pooled animals were used until chalimus II stage (ca. 100 animals in free-living stages, ca.10 for chalimi), and for the later stages, individual animals were used. Three samples per stage were analyzed.

To find out if the expression of the genes of interest fluctuates during egg-string development, more than 50 adult females were collected from fish, their egg strings were removed and transferred into hatching wells, and the females from which the egg strings were removed were directly put into RNAlater for later gene-expression analysis. The egg strings were observed for the following 10 days, at least once a day, and their hatching time was noted. Based on the known time of sampling and the hatching time, the progress of each female throughout the egg-string cycle could be calculated. Three random samples for 10 time intervals (0–1 days till hatch to 9–10 days till hatch) were selected, and the RNA was isolated from whole adult females (without the egg string) and used for qPCR of the genes of interest. The relative expression was normalized to the average expression of all samples for each gene.

To determine efficiencies of RNAi knockdown (see Section 2.7), RNA from five to six control animals, as well as experimental animals, was isolated. The relative expression of the experimental animals in comparison to the control animals was determined, using the 2^−ΔΔCT^-method [22], employing *EF1A* and *ADT3* as reference genes, as described above. All genes of interest in this study were measured for each treatment.

For the analysis of knockdown interactions, we reused samples (4 knockdown samples and 4 controls) that had been created for the analysis of Rheb [11] and the TOR-pathway [10].

### 2.6. In Situ Hybridization

We used an established protocol for in situ hybridization on salmon lice, as described before [23]. Animals were fixed in 4% PFA, embedded in paraffin, and cut into 5 µm thick sections. DIG-labeled probes were generated, using the DIG RNA Labeling kit (Roche, Basel, Switzerland), and hybridized to the sections at 65 °C. For every gene an antisense probe, which should give specific positive signal and a sense probe, which was used as negative control, were generated. Chromogenesis was performed with the BCIP/NBT Liquid Substrate System (Sigma-Aldrich, St. Louis, USA).

### 2.7. RNAi Knockdown

RNAi knockdown was performed as described before [9]. Double-stranded RNA was produced by using the MEGAscript RNAi kit (Ambion, Austin, USA). A sequence coding for cod trypsin was used as negative control. Double-stranded RNA (ca. 0.5 µl of a 600 ng/µl solution) was injected into preadult II females, with fine-glass capillaries. The animals could then recover for at least one hour in seawater, before they were put on an Atlantic salmon. Then, 10 females and 10 males were set together on a fish. For each experiment, three fish were used (more for the controls, due to several dates of the experiments). Lice were collected five weeks after injection and photographed, and egg strings were collected and put into hatching wells. The females were placed in RNAlater, for analysis of the knockdown efficiency (see Section 2.5). Five weeks after injections, lice were expected to have developed into adult females, carrying their second pair of egg strings. An efficient gene knockdown in salmon louse was demonstrated for at least 69 days after injection [24]. Several animals were also fixed in Karnovsky fixative, for further sectioning and histological analysis.

### 2.8. Histological Analysis

Animals for histology were fixed in Karnovsky fixative and kept at 4 °C, until processing. Animals were embedded in Technovit 7100 (Kulzer, Hanau, Germany) and sectioned with a thickness of 2 μm. Staining was performed by immersing the slides in a 1% Toluidine blue, 2% borax solution for 1 min, followed by a wash in running tap water, until the background was cleared. Slides were air-dried and mounted with DPX new (Merck, Darmstadt, Germany). Three animals per condition were analyzed.

### 2.9. Protein Analysis

Cement glands were dissected from adult female lice and opened by cutting off the tip with a scalpel blade. The content of the gland was removed by pulling it with forceps through the opening while holding the gland with a second set of forceps at the other end. The extracted substance was put into RIPA buffer containing protease inhibitors and was smashed on ice with a pestle, in a reaction tube. The solution was centrifuged at max speed at 4 °C for 20 min. The supernatant was removed, and the pellet was dissolved in an 8 M Urea buffer containing 1% 2-mercaptoethanol. The procedure with pestle and centrifuge was repeated again, and the supernatant was used for protein separation on a gel. We used stain-free 4%–15% TGX gels (Bio-Rad, Hercules, USA). Samples were combined with a loading buffer (Pierce Lane Marker Reducing Sample Buffer, Thermo Scientific) and applied to the gel, and the gels were run with the recommended conditions. Gels were stained with Colloidal Coomassie (Bio-Rad, Hercules, USA) and photographed. Bands of interest were cut out with sterile scalpel blades and used for mass spectrometric analysis. Mass spectrometry was performed by the Proteomics Unit at the University of Bergen (PROBE). In brief, the samples were digested in-gel with trypsin, and the peptides were extracted, desalted, and then applied to an Orbitrap Elite LC–MS/MS mass spectrometer.

Analysis of the mass spectrometric results was performed, using ProteoWizard [25], SearchGUI 3.3.11 [26], and PeptideShaker 1.16.36 [27], using the current Licebase protein database for salmon lice, with the additional sequences from this article.

## 3. Results

In the Licebase gene-expression data, we found fourteen genes fulfilling the filtering criteria for genes expressed inside the genital segment, but not in extruded egg strings (Table S2). Blasting identified five of them as Hemicentins, one as a Netrin receptor, one as Teneurin-a, one as Tenascin-X, one as Delta-like protein C, and two as Chorion peroxidases. As the hits were not of very high quality, and thereby the annotation was dubious, we continued with a more thorough analysis of the gene and protein structure.

### 3.1. Gene Structure

We performed conventional PCR, as well as RACE-PCR, to verify the gene sequences from Licebase/EnsemblMetazoa and to obtain the complete CDS of each gene. In this process, we found out that the annotation of these predicted genes in the LSalAtl2s assembly is partly flawed. Some exon–intron structures, as well as coding domain starts, were wrongly predicted, and one gene was separated and distributed onto several scaffolds. We could overall identify nine novel genes (Table 1) that have not been described before. We assigned new gene names based on the characteristics that we identified in this study (FCGS1-6, FCGMB1, and FCGPO1-2).

Most of the genes encoded secreted proteins, as indicated by the presence of signal peptides. Another protein contained an N-terminal transmembrane-domain, and one of the peroxidases contained a C-terminal transmembrane domain.

**Figure 2:**
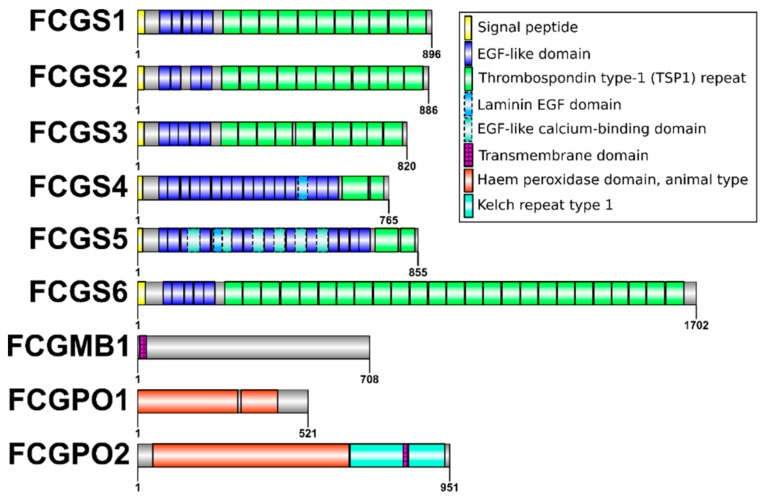
Protein domain structure. Domains were predicted, using InterProScan [18]. The graph was prepared, using IBS [28].

An overview over the domain structure of the proteins is given in Figure 2. All FCGSs share the presence of a signal peptide, several epidermal growth factor (EGF)-like domains, and several thrombospondin 1 (TSP1)-like repeats. The domain structures of FCGS1, FCGS2, and FCGS3 are quite similar, with 4–5 EGF domains and 10–11 TSP1 repeats. FCGS4 has an increased number of EGF-domains (17) and only two TSP1 repeat domains. FCGS5 has 20 EGF domains and only two TSP1 repeats. Additionally, six calcium-binding and one Laminin EGF-domain were detected. FCGS6 is more than twice as long as the other proteins, and this is mainly based on additional TSP1 repeats (overall 25).

During sequence analysis, we noticed an unexpectedly high cysteine content for the encoded proteins (Table 2). Taking the average of all annotated sea louse proteins, the average cysteine content is 2.1%. In contrast, FCGS1-5 and FCGMB1 had five times as many cysteine residues. In particular, FCGS5, with a cysteine content of 16.3%, was striking, having the 12th highest cysteine content of all predicted salmon louse proteins.

We identified homologues to the FCGSs in several other copepod species (Table S3). These were detected in other members of the Siphonostomatoida (*Caligus rogerecressey* and *Tracheliastes polycolpus*) and in cyclopoids (*Eucyclops serrulatus* and *Paracyclopina nana*). Prediction of the open reading frames with subsequent translation into putative proteins revealed the typical presence of EGF-domains and TSP1-repeats.

### 3.2. Gene Localization

**Figure 3:**
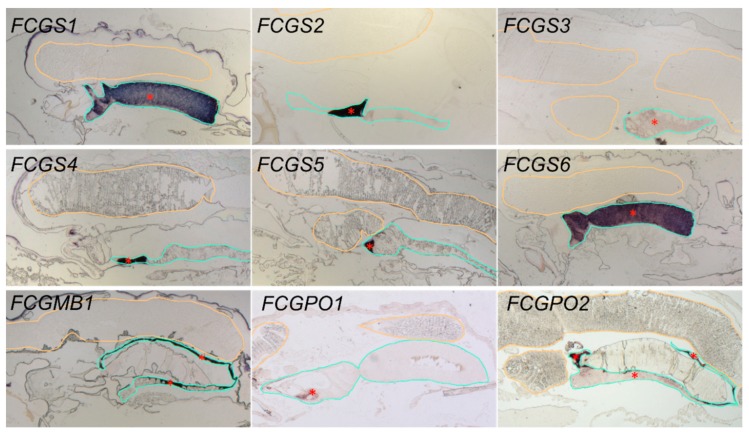
Localization of the transcripts of the GOIs in an adult female salmon louse, as determined by in situ hybridization. Signal was only obtained in the genital segment of the lice. Every image shows only one-half of the genital segment; similar signal was obtained in the other half. The developing eggs found in the genital segment are highlighted with orange, for better visibility; the cement gland is circled in turquoise. Specific staining is marked with red asterisks (*). Negative controls (sense probes) did not give signals in that region. For an overview of the complete animal and a negative control, see Figure S1.

In situ hybridization was performed in order to locate the transcripts within the female salmon louse. For all the genes, a positive signal was only found in the cement glands and not in the developing eggs (Figure 3). The expression patterns were very distinct for the different genes. *FCGS1* and *FCGS6* were strongly expressed throughout the whole length of the cement gland. *FCGS3* gave a much weaker signal but could be observed throughout the cement gland. *FCGS2*, *FCGS4,* and *FCGS5* expression, on the other hand, were clearly limited to small regions of the cement gland, distal in the genital segment, close to the genital antra. *FCGMB1* had a specific expression profile on its own, and its signal was found only in the inner layers of the cement gland that were very close to the cement filling. Both peroxidases had distinct expression patterns. While the expression of *FCGPO2* resembled the expression of *FCGMB1*, with an expression close to the lining of the cement inside the gland, *FCGPO1* showed a specific patchy expression in some cells, at the most posterior part of the cement gland.

### 3.3. Gene Expression

**Figure 4:**
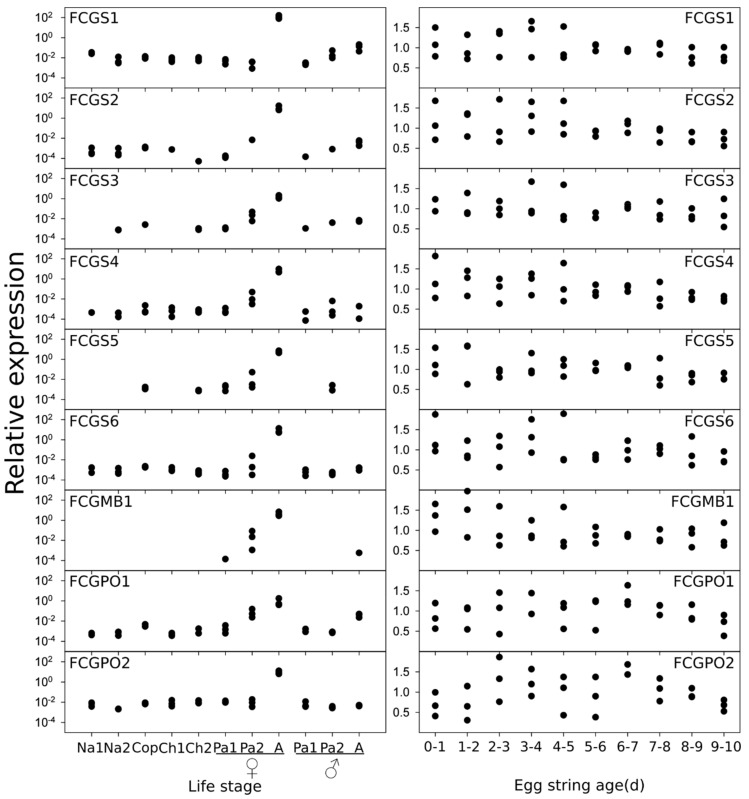
Gene-expression profiles. The left-hand panel shows the relative gene expression of the different GOIs in different life stages of *L. salmonis* on a logarithmic scale. Na = Nauplius; Cop = Copepodid; CH = Chalimus; Pa = Preadult; A = Adult. Starting from the preadult stage, females and males were measured separately. The right-hand panel shows the relative expression of the GOIs in the mothers during the egg-string production cycle normalized to the average expression of each gene in all samples. A high egg-string age indicates that a hatching is occurring soon. Each dot represents an individual sample.

We used qPCR to verify the expression of the genes in different life stages of salmon lice, as well as during the different stages of egg-string development (Figure 4). All analyzed genes were found to be expressed strongest in adult females and some to a much lower extent in preadult II females and at negligible levels in males or earlier stages. Expression levels were comparable between the different genes. The only exception was *FCGS1*, which had a tenfold higher expression than the other genes. During the egg-string developmental cycle, no changes in gene expression were obvious, the expression of the genes seemed to remain stable during all ten days of egg string development (Figure 4).

### 3.4. RNAi-Mediated Knockdown

**Figure 5:**
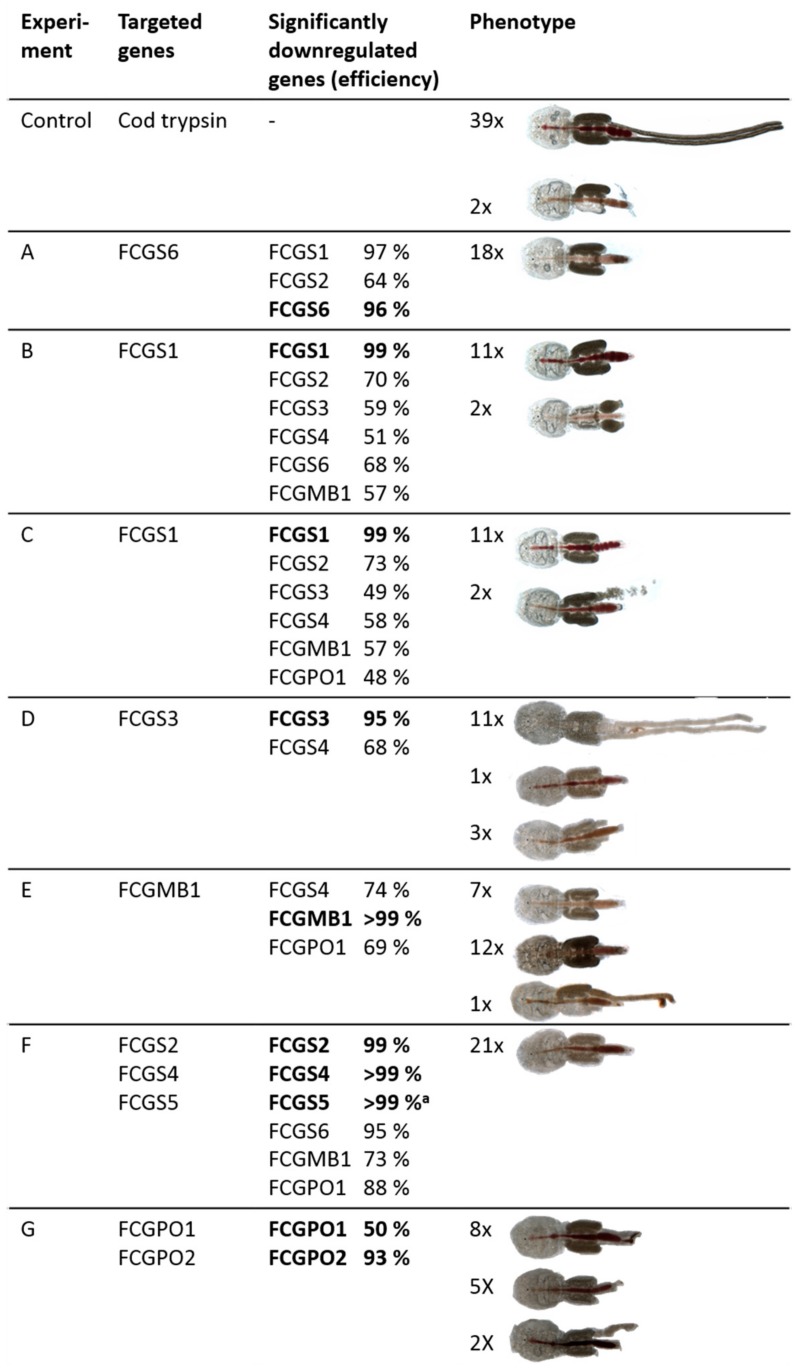
RNAi knockdown efficiencies and resulting phenotypes. In each experiment, 30 preadult female salmon lice were injected with double-stranded RNA, targeting the genes given in the second column. Downregulation of all genes of interest of the cement gland was assessed (third column). The fourth column gives the number of lice with a certain phenotype, collected from fish. The control experiment was performed several times, explaining the higher numbers in the phenotype column for the controls. Significantly downregulated genes were determined by Welch’s t-test, taking into account multiple testing of all genes in the individual experiments, using Bonferroni correction (*p*adj < 0.05). ^a^ Not significant, but mentioned as one of the targeted genes.

To evaluate the function of the genes that we found in the cement gland, we employed RNAi-mediated knockdown of these genes in preadult II females. Seven knockdown experiments were performed, targeting different genes of the cement gland (Figure 5). Validation of the knockdown efficiency revealed that all of the double-stranded RNAs used for the knockdown gave a downregulation of several genes. The knockdowns of the targets were highly efficient, with a reduction of the target mRNA levels by over 94%.

Furthermore, in every experiment, apart from experiment G, the knockdown of at least one nontargeted gene was also observed. Two different dsRNAs targeting *FCGS1* (experiment B and C) also had significant effects on a different number of other genes. Assessing the phenotypes, the importance of the genes for the egg-string development became clearly apparent. In animals that had been injected with a control dsRNA not targeting any known sea louse gene, all animals (but two) produced egg strings and carried them attached to their body. On the contrary, after knockdown of individual or several *FCGS*s, egg strings were either completely absent or malformed. In experiment A, which led to a very strong downregulation of *FCGS1* and *FCGS6*, all females were missing any form of egg string. In experiment B, with a very strong downregulation of *FCGS1* and weaker downregulation of three other *FCGS*s, as well as *FCGMB1*, most animals were missing egg strings, and, in two cases, the egg strings had a balloon-like shape (Figure 5, experiment B, second picture). In experiment C, most animals were again missing egg strings, and only two animals were carrying a malformed, shortened egg string with eggs placed in an apparently random pattern. In experiment D, targeting *FCGS3*, also reducing levels of *FCGS4*, most animals produced egg strings; however, they were curly (Figure 5; experiment D, first picture). In experiment E, *FCGMB1* was knocked down to very low levels. *FCGS*4 was downregulated, as well, in addition to *FCGPO1*. Most of the animals were carrying very short threads of cement attached to their genital openings; the other lice (except one) were neither carrying egg strings nor cement. In experiment F, four *FCGS*s, as well as *FCGMB1* and one peroxidase, were downregulated to different levels. All of the animals were completely lacking egg strings. Experiment G targeted the two peroxidases, *FGCGPO1* and *FCGPO2,* only. The phenotypes showed severe malfunctions in egg-string synthesis. In most of the animals, some form of egg string could be observed, seemingly only consisting of the outer material and not containing any eggs at all. In several other animals, even this structure was absent. Only in two animals, eggs in egg strings could be observed; however they were not organized and stacked as they were in the controls.

**Figure 6:**
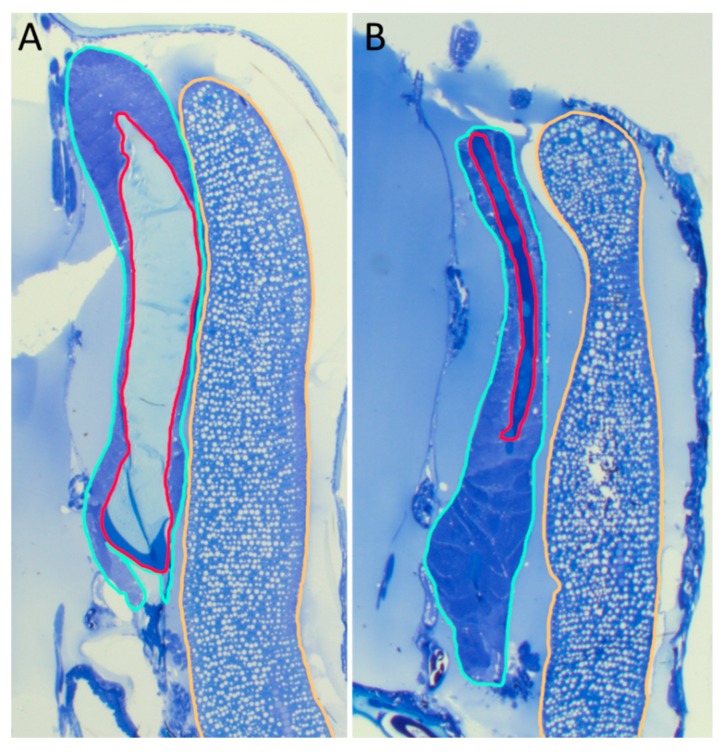
RNAi knockdown effects on cement gland content. (**A**) Control animal. (**B**) Animals with a knockdown targeting *FCGS2*, *FCGS4,* and *FCGS5* (experiment F, cf. Figure 5), leading to a downregulation of most of the analyzed genes (cf. Figure 7A). Representative sections of the slides with the thickest layers of cement observed. One-half of the genital segment of an animal is shown. Maturing eggs are circled in orange, cement glands in turquoise, and cement in red.

Animals from the RNAi experiment were sectioned and stained in order to further examine the phenotype (Figure 6). In experiment F, where six genes of interest were downregulated, the cement glands were almost completely empty. In the control animals, the cement gland was almost completely filled with light blue staining cement (Figure 6A), whereas, in the knockdown animals, only a very thin line of cement that was stained in a darker blue was present (Figure 6B). Developing eggs, on the other hand, were present in controls, as well as knockdowns, and did look well organized in either group.

### 3.5. RNAi Knockdown Interactions

**Figure 7:**
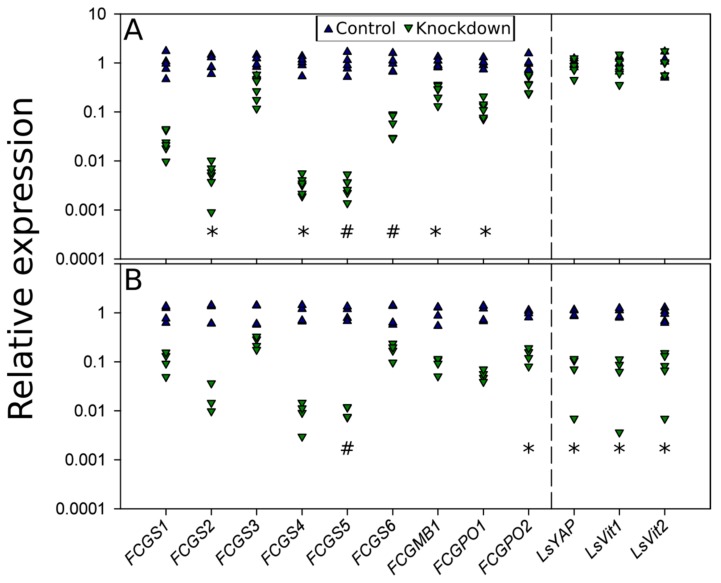
Gene expression levels after RNAi knockdown. (**A**) Knockdown of *FCGS2*, *FCGS4,* and *FCGS5* (Experiment F, compare with Figure 5); (**B**) knockdown of Ras homolog enriched in brain (*RHEB*). Asterisks (* *p*adj < 0.05) and hash (#, *p*adj < 0.1) mark significant differences between controls and knockdowns, after accounting for multiple testing. Each triangle represents the expression of one individual.

Since knockdown of individual cement-gland genes gave knockdown of others, we examined if this knockdown could affect the egg maturation in the genital segment by influencing genes involved in this process. Additionally, we examined if knockdowns of genes known to have an effect on egg maturation affect cement gland genes (Figure 7). The knockdown of *FCGS2*, *FCGS4,* and *FCGS5* led to the knockdown of most analyzed genes of the cement gland. Important egg maturation genes (LsYAP, LsVit1, and LsVit2), on the other hand, were not affected by this knockdown. The knockdown of *RHEB* led to a downregulation of the vitellogenins and *LSYAP,* as well as *FCGPO2* and *FCGS5*. While not being statistically significant, due to the multitude of tests, the other cement gland genes showed a clear trend toward downregulation. A knockdown of the ecdysone receptor that is known to lead to a downregulation of LsYAP and vitellogenins did not lead to significant changes in the expression of cement-gland genes.

### 3.6. Protein Analysis

**Figure 8:**
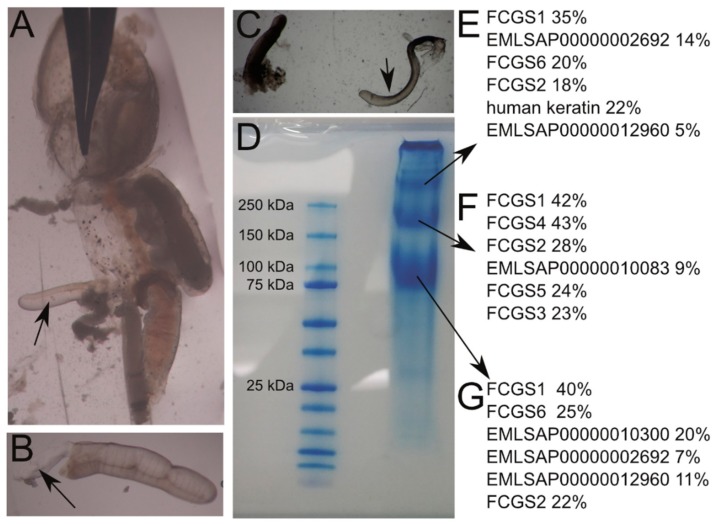
Protein isolation and identification from the cement of a salmon louse. (**A**) The genital segment of a salmon louse was cut open laterally, and the developing eggs were removed, so that the cement gland (arrow) became accessible. (**B**) The cement gland was separated from the rest of the animal. At the cutting site, cement (arrow) is leaving the gland. (**C**) The cement (arrow) can be drawn in one thread-like piece out of the gland. (**D**) SDS-PAGE gel of the cement. (**E**–**G**) Results of mass-spectrometric analysis of the cut-out bands as indicated by the arrows in (**D**). Top 6 hits together with the relative coverage of each protein by the detected peptides.

As we found *FCGS*-genes expressed in the cement-gland tissue and encoding proteins with signal peptides, we wanted to test if these proteins are part of the cement itself. To this end, we dissected the cement glands from adult female lice and isolated the cement from the glands (Figure 8). It was possible to extract the cement like a thread from the gland, and it showed some glue-like properties. While it was well shaped directly after removal from the gland, movement with forceps in the water lead to the loss of any recognizable structure, and it was impossible to fold it back into its original form. Protein isolation in a urea buffer and subsequent separation in a denaturing SDS gel led to the identification of several clear, strong bands in the gel (Figure 8). The smallest protein had a molecular weight between 75 and 100 kDa, the next had a size of around 200 kDa, and the largest protein had a molecular weight of over 250 kDa. It also became apparent that several proteins had barely entered the gel and had not migrated, as indicated by a band directly on top of the gel, very close to the wells. The three main bands were cut out of the gel and analyzed with mass spectrometry. For every band, the best hit (high coverage and high number of spectra) was FCGS1, and all the other members of the FCGS family were identified in at least one of the bands. Additional hits were vitellogenins (EMLSAP00000012960, EMLSAP00000010300, and EMLSAP00000010083), a hemocytin (EMLSAP00000002692), and a SCO-spondin (EMLSAP00000002692). Some contamination with human keratin, which must have occurred during processing of the samples, was observed. Several attempts to isolate proteins from an empty egg string after hatching were not successful (not shown).

## 4. Discussion

### 4.1. FCGS Are the Building Blocks of the Egg Strings

Overall, our results clearly indicate that the newly identified FCGS-proteins are the major component of the egg string of the salmon louse. It was established more than a hundred years ago that the cement gland’s cement is used to make the egg string [29]. We could show that the genes that encode the FCGS proteins are highly specific for the cement glands of females, as shown by sex- and stage-specific qPCR, as well as in situ hybridization in this work. They all contain a signal peptide, indicating their secretion. Additionally, we could verify the presence of FCGS1-6 in the cement on the protein level. Knockdown of the genes led to the absence of cement in the cement gland and prevented the development of egg strings.

### 4.2. FCGS Forms a New Protein Class

Blast results suggested that FCGS1-6 belongs to the hemicentin-family. Hemicentins are evolutionary old proteins of the extracellular matrix, important for, among other things, cell migration [30]. However, a detailed analysis shows that the FCGS-gene products—in spite of the homology—are not true hemicentins. Key characteristics of hemicentins are the presence of a von Willebrand/Integrin A domain and a series of immunoglobulin modules [31]. As a subgroup of the fibulin-family, they also have EGF-domains at their C-terminal end. The human hemicentin-1 also has several thrombospondin repeats [31]. FCGS1-6 consist only of EGF-domains and thrombospondin repeats and thereby do not fulfil the hemicentin criteria. The presence of several thrombospondin-like repeats might alternatively suggest that the proteins are thrombospondins. However, this is also not the case. Key characteristics of thrombospondins are the presence of several EGF domains and several thrombospondin type-3 repeats, as well as L-type lectin-like domain [32], criteria not fulfilled by the salmon louse proteins. Overall, the FCGS-proteins seem to be part of a new, formerly unknown protein-family, as a search in InterProScan for proteins with a similar structure did not yield any results. 

However, our search for homologous sequences in the transcriptome shotgun assemblies of other arthropods revealed that FCGS proteins are not unique for salmon lice; they are also present in other copepod species of the orders Siphonostomatoida and Cyclopoida. In the assemblies of *Calanus finmarchicus*, *Eurytemora affinis,* and other Calanoids, no homologues were found, suggesting that the use of FCGS proteins for egg-string formation is specific for Siphonostomatoids and Cyclopoids. This feature seems thereby to be less conserved than the genes involved in the synthesis of the wall of the spermatophores of males. These have been found in Calanoida, as well [23].

### 4.3. Genes of the Cement Gland Are Co-Regulated

The results of the knockdown experiments suggest a co-regulation of the genes of the cement gland. In every experiment, we found the downregulation of genes that have not been targeted by the double-stranded RNA. In some case, this might be explained by high homology: The *FCGS*-genes all encode the same protein domains, so a high similarity is clear. Just a small overlap between dsRNA and a nontargeted gene can lead to off-target effects [33]. On the other hand, in several experiments, the targeted knockdown of only *FCGS*-genes also led to the downregulation of *FCGMB1* and, in one case, also of a peroxidase. The knockdown of *FCGMB1* also led to a significant knockdown of *FCGS4* and *FCGPO1*. Neither *FCGMB1* nor the two peroxidases share any domains with *FCGS*-proteins; thereby, a pure off-target effect seems unlikely. This is even more the case since the three genes, *LsYAP*, *LsVit1,* and *LsVit2,* were not downregulated, either, in an experiment where the knockdown of three *FCGS*-genes led to the downregulation of most analyzed cement gland genes. Vitellogenins [7] and LSYAP [9] are important for the development of the eggs in the genital segment of the louse. This seems to be independent from the actual cement synthesis. The expression of the genes was unaffected by the knockdown, and also, histologically, it became apparent that even with almost empty cement glands, normal-looking eggs were produced in the genital segment. On the other hand, the knockdown of a gene that lead to a downregulation of the vitellogenins also had an effect on the cement-gland genes. A downregulation of cement-gland genes was observed after the knockdown of *Rheb*. Rheb is part of the TOR pathway and has been shown to be important for egg maturation in the sea louse [10]. Cement production might thereby be under the control of the TOR pathway.

The result that the genes producing the cement and thereby the egg strings are expressed constantly at same levels in adult females was surprising. We assumed that there might be variation. Directly after the extrusion of the egg string, there might be a higher demand for the production of new cement, and thereby the expression might be increased. On the other hand, this result is in line with former findings. A cyclical expression in line with the reproductive cycle had been assumed for vitellogenin; however, such an expression pattern could not be found [7].

FCGS1 expression was analyzed on a microarray before, with two probes, targeting “Contig 12” and “Contig 202”. The analysis [34] showed that this gene is increasing in expression, not directly at the beginning of the adult stage, but in the “T3 stage”, a later stage in which, after some post-moulting growth, oocytes can be seen in the genital segment. According to a Northern blot, there was no signal before this stage. In contrast, vitellogenins 1 and 2 are firstly expressed in the “T4 stage”, indicating that cement production starts before vitellogenenesis.

### 4.4. FCGMB1 and FCGPO1 and 2 Are Essential for Egg String Formation

Results from the knockdown of FCGMB1 clearly showed that the encoded protein is involved in the egg-string formation. However, its actual function remains unclear, as it does not contain any conserved domains. It was not identified by proteome analysis in the cement. This fits with the fact that it does not contain a signal peptide, but a transmembrane domain. This is located N-terminally, so most of the protein probably sticks out of the membrane of the cells of the cement gland, closest to the lumen of the gland. This was confirmed by the result of the in situ hybridization, where only a thin line, along the gland’s lumen, showed signal for this gene.

The role of the two peroxidases, FCGPO1 and FCGPO2, seems clearer. Peroxidases are capable of cross-linking proteins [35]; for example, they can form intermolecular dityrosine bonds [36]. We assume that the peroxidases cross-link the various FCGS proteins to form the cement. This might also explain why we were incapable of isolating individual proteins from extruded egg strings, as well as the banding pattern of the cement proteins in SDS page. In spite of using SDS, 2-mercaptoethanol, and DTT, three major bands were identified, in each of which for example FCGS1, together with the other FCGS proteins, was identified. This suggests some strong covalent bonds between the proteins that could not be destroyed using the employed conditions. Additionally, we assume that the proteins are heavily relying on disulphide bonds for their structure, due to the extraordinarily high amount of cysteine in the proteins. 

## 5. Conclusions

We have identified six proteins that are building blocks for egg strings, as well as three proteins that are necessary for the egg string synthesis in *L. salmonis*. Similar genes are present in the genomes of only relatively closely related copepods. By preventing the cement production, one might prevent the generation of egg strings and thereby the reproduction of salmon lice in aquaculture.

## Figures and Tables

**Table 1:** GOIs and their characteristics.

Gene Symbol	Gene Name	ENSEMBL Stable Gene id(s)	GenBank Accession	Signal Peptide	Trans-Membrane Domain	mRNA Length (bp) ^1^	CDS Length (bp)/Protein Length (aa)/Protein Size (kDa)
FCGS1	Female cement gland secreted 1	EMLSAG00000005417 EMLSAG00000002486 EMLSAG00000002488 EMLSAG00000002798 EMLSAG00000009842	MK933815	+	-	2811	2691/896/97
FCGS2	Female cement gland secreted 2	EMLSAG00000002328	MK933816	+	-	2801	2661/886/97
FCGS3	Female cement gland secreted 3	EMLSAG00000008518	MK933817	+	-	2564	2463/820/92
FCGS4	Female cement gland secreted 4	EMLSAG00000011195	MK933818	+	-	2423	2298/765/84
FCGS5	Female cement gland secreted 5	EMLSAG00000010070	MK933819	+	-	2632	2568/855/94
FCGS6	Female cement gland secreted 6	EMLSAG00000010397	MK933820	+	-	5501	5109/1702/186
FCGMB1	Female cement gland membrane bound 1	EMLSAG00000000454	MK933821	−	N-terminal	2334	2127/708/77
FCGPO1	Female cement gland peroxidase 1	EMLSAG00000007125	MK933822	−	-	1884	1566/521/60
FCGPO2	Female cement gland peroxidase 2	EMLSAG00000010106	MK933823	−	C-terminal	3303	2856/951/109

^1^ Based on RACE, without Poly-A-tail.

**Table 2:** Cysteine contents of the proteins of interest and the average of sea lice proteins.

Gene Symbol	Cysteine Content
FCGS1	11.7%
FCGS2	11.9%
FCGS3	11.8%
FCGS4	15.8%
FCGS5	16.3%
FCGS6	11.2%
FCGMB1	11.7%
FCGPO1	1.7%
FCGPO2	2.7%
⌀ *L. salmonis* proteins	2.1%

## Supplementary Materials

The following are available online at https://www.mdpi.com/2073-4425/10/12/1004/s1. Table S1: Primers used in this study. Table S2: Genes fulfilling the filtering criteria, and their CPM values in different early stages, as well as the Swiss-Prot hit with the lowest e-value. Table S3: Accession numbers of sequences from NCBI’s transcriptome shotgun assembly database, potentially encoding homologues of FCGSs based on the presence of EGF and TSP-domains. Figure S1: Complete louse sections after in situ hybridization for FCGS6. Negative control (sense probe) on the left-hand side, antisense probe on the right-hand side. Unspecific staining seen in both sections in cuticle and tissue folds. Specific staining visible in cement gland with antisense probe. The red rectangle marks the section regions shown in Figure 3. Uneven light conditions in the pictures are due to manual stitching of two individual photos per slide, to get the complete animal in one picture.
