# Supplementary material for: Identification and Description of the Key Molecular Components of the Egg Strings of the Salmon Louse (Lepeophtheirus salmonis)
Source: Genes (Basel). 2019 Dec 3;10(12):1004. doi: 10.3390/genes10121004 (PMC6947537; doi:10.3390/genes10121004)
Supplement: Supplementary file 1 [file genes-10-01004-s001.zip › in-situ-supplement2.pdf]

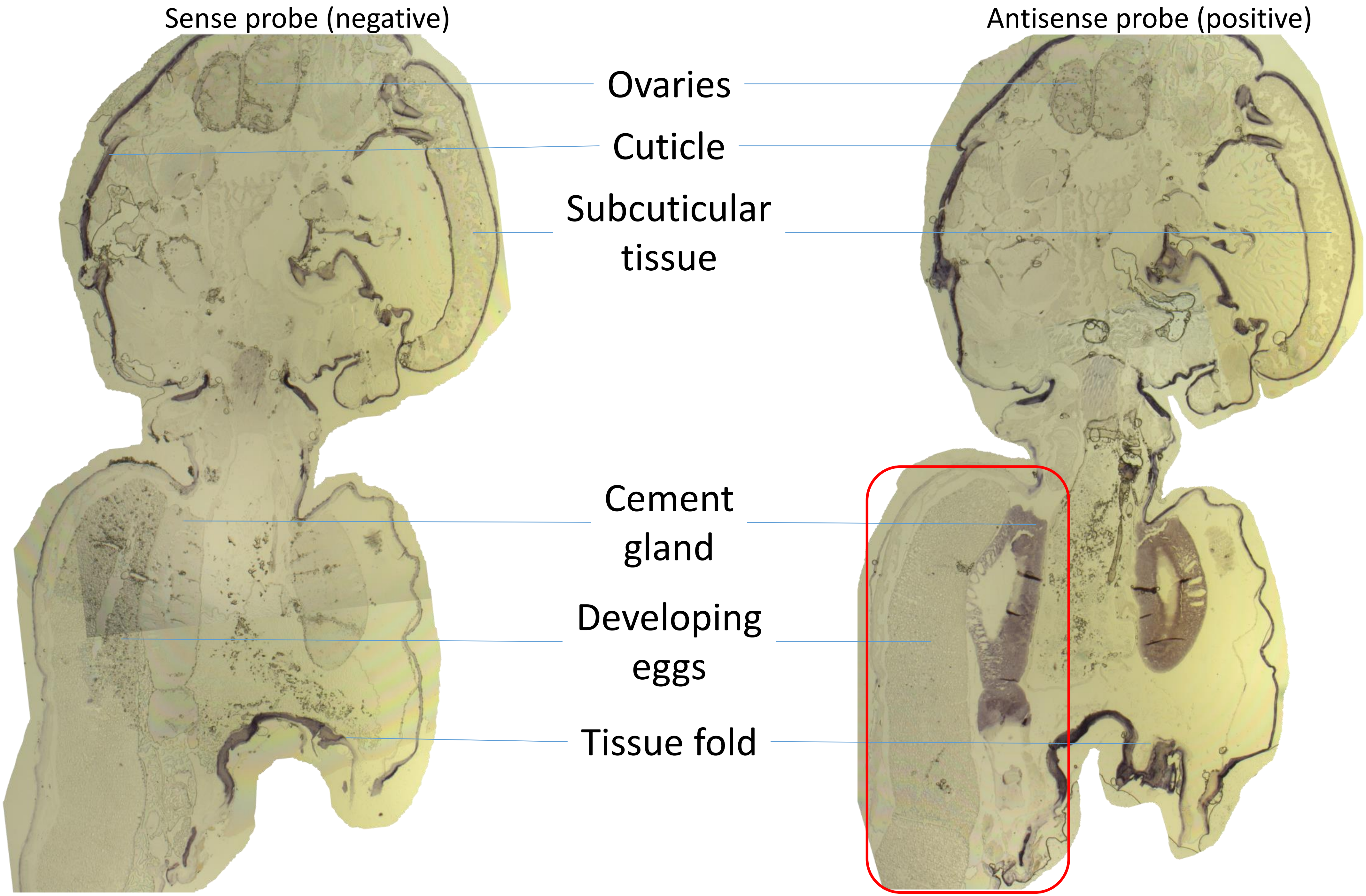

Fig. S1: Complete louse sections after in situ hybridization for *FCGS6.* Negative control (sense probe) on the left hand side, antisense probe on the right hand side. Unspecific staining seen in both sections in cuticle and tissue folds. Specific staining visible in cement gland with antisense probe. The red rectangle marks the section regions shown in Fig. 3. Uneven light conditions in the pictures are due to manual stitching of two individual photos per slide to get the complete animal in one picture.
